# Perioperative Management of Anesthesia in Patients With Cardiovascular Disease: A Review of Current Guidelines in the United States

**DOI:** 10.7759/cureus.79355

**Published:** 2025-02-20

**Authors:** Doncollins Okolo, Wisdom S Ugorji, Nenrot S Gopep, Chika C Oragui, Chioma C Ubajaka, Okelue E Okobi

**Affiliations:** 1 Anesthesiology, St. George's University's School of Medicine, Maywood, USA; 2 General Practice, NHS England, Newcastle Upon Tyne, GBR; 3 General Practice, Fell Tower Medical Centre, Newcastle Upon Tyne, GBR; 4 Community Medicine, Federal Medical Center, Keffi, NGA; 5 Public Health, Georgia Southern University, Statesboro, USA; 6 Pediatrics/Pediatric Intensive Care Unit (PICU), Stanford University School of Medicine, Lucile Packard Children's Hospital, Palo Alto, USA; 7 Internal Medicine, Igbinedion University Okada, Benin City , NGA; 8 Family Medicine, Medficient Health Systems, Laurel, USA; 9 Family Medicine, Lakeside Medical Center, Belle Glade, USA; 10 Family Medicine, Larkin Community Hospital Palm Springs Campus, Miami, USA

**Keywords:** anesthesia, cardiac surgical procedures, cardiovascular disease, perioperative, perioperative management

## Abstract

Perioperative anesthesia management in cardiovascular disease (CVD) patients is an important and multifaceted process requiring careful planning, multidisciplinary coordination, and keen observance of evidence-based guidelines. Thus, owing to the increased incidence rate of perioperative complications, including postoperative and intraoperative hemodynamic instability, arrhythmias, and ischemic events, CVD patients are considered a representative group for comprehension of the broader influence of anesthesia on cardiovascular function. Anesthetic methods and agents affect hemodynamic stability and poor management might result in exacerbated complications. Therefore, the objective of this review is to evaluate and summarize the United States' guidelines on perioperative management of anesthesia in CVD patients, highlighting the best anesthesia practices, strategies for risk assessment, and evidence-based recommendations for optimizing patient outcomes and reducing perioperative complications. The research question the study seeks to answer is how the existing U.S. guidelines inform the perioperative management of anesthesia in CVD patients and what evidence-based interventions are proposed for the optimization of patient outcomes and reduction of perioperative complications. The study has reviewed guidelines developed by the American College of Cardiology (ACC) and the American Heart Association (AHA), which focus on aspects of perioperative anesthesia management, including perioperative risk assessment, hemodynamic management, and anesthetic techniques. The literature search was conducted on different online databases, including PubMed, Scopus, Web of Science, and Google Scholar, for published and peer-reviewed literature that focused on perioperative anesthesia management and guidelines in the United States. Moreover, the search strategy utilized the Preferred Reporting Items for Systematic Reviews and Meta-Analyses (PRISMA) guidelines in the selection and subsequent inclusion of articles for the review. The systematic review has disclosed that anesthesia is more than the observation of an unconscious patient, as the four key anesthesia components should be provided and managed with care, owing to anesthesia’s potential adverse effects. While there is no universal agreement on the provision, development, and use of a general protocol, careful consideration of every type of anesthesia and the related effects on patients with CVD is important to the provision of safer and proper care.

## Introduction and background

Patients with cardiovascular disease (CVD) often present various challenges with regard to perioperative anesthetic management, and this has been attributed to the possible risks that include myocardial injury, arrhythmias, and hemodynamic instability. Studies focusing on perioperative and anesthesia-associated mortality have indicated global improvements with regard to CVD patient outcomes in the last five decades [1]. Notably, general mortality attributable to anesthesia has significantly reduced from approximately 357 per 1,000,000 patients before the 1970s to approximately 34 per 1,000,000 patients, regardless of the increase in the overall number of patients having higher anesthetic risks [1, 2]. Such reductions are attributable to improved anesthetic agents with improved hemodynamic stability, refined perioperative care protocols, and improved monitoring methods, including anesthesia depth monitoring and transesophageal echocardiography, which have enabled timely detection and management of potential complications that include myocardial ischemia and arrhythmias.

Furthermore, studies have shown a positive correlation between anesthetic mortality and the Human Development Index (HDI), which is linked to income, education, and life expectancy [1]. In nations such as the United States that have higher HDIs, anesthesia-related mortality has reduced from approximately 357 per 1,000,000 patients to approximately 25 per 1,000,000 patients [1]. Based on this, various American guidelines have emphasized a customized approach that balances the requirement for sufficient anesthesia and mitigation of potential cardiovascular risks [3, 4]. However, at present, no single universally approved anesthesia management technique for CVD patients exists [5]. Rather, medications and various combinations of drugs are employed based on aspects that include the anesthesiologist’s experience, the patient’s individual preferences, and the patient’s pathophysiological state [5]. The provision of safe anesthesia to CVD patients has often been a major challenge. The combined effects of different anesthetic drugs, alongside the surgical procedure’s physiologic stresses, and the underlying CVD have the potential to not only complicate but additionally restrict the options for anesthetic techniques used for specific procedures [6, 7]. Normally, the approach employed by anesthesiologists in relation to CVD patients entails the selection of techniques and agents that optimize the CVD patient’s cardiopulmonary functions [6]. Thus, perioperative CVD patient management calls for closer cooperation between the surgeon, anesthesiologist, cardiologist, interventionist, and intensivist, given that every specialist has an exceptional knowledge base complementing others for better care outcomes. Such a multidisciplinary team approach must emphasize the importance of the care continuum, beginning with the preoperative assessment to the postoperative period. Thus, given the increase in complexity of CVD patients who required surgical interventions in the last decades, the emergence of concepts such as hospital “Heart Teams” has been witnessed [7]. Precisely, such multidisciplinary teams are not only tasked with decision-making but also with choosing the most appropriate optimal treatment approach for every patient [7-11]. Moreover, the team approach has been successfully applied in different medical fields, including organ transplantation medicine and oncology, and significant improvements with regard to care quality have been reported. Such concepts have been widely emphasized in the CVD patient care context, thereby making them standards for CVD care globally [12-15].

Consequently, guidelines that include the American Heart Association and the American College of Cardiology (AHA/ACC) have offered perioperative cardiovascular assessment guidelines in non-cardiac surgeries, evaluating risks based on clinical markers, type of surgery, and functional capacity [16-20]. Such guidelines, which mainly draw on expert opinions and observational studies [21], have also suggested non-invasive cardiac stress assessment for high-risk patients who have multiple cardiac risk factors and are undergoing vascular surgeries, potential myocardial revascularization, and coronary angiography with high-risk test outcomes [22-27]. Furthermore, perioperative acute ischemic stroke (PAIS) is still a serious complication affecting even patients without prior cerebrovascular disease and has been attributed to perioperative thromboembolism, hemodynamic instability, and hypercoagulability, resulting in considerable mortality and morbidity [28]. Existing evidence has indicated that perioperative statin therapy, notwithstanding the baseline lipid status of the cardiac surgery patient, has the potential to reduce mid-term mortality while also exerting protective effects beyond lipid reduction [29-33]. Based on the above observations, the objective of this study is to review and summarize current U.S. guidelines on perioperative anesthesia management in CVD patients, highlighting the best practices, strategies for risk assessment, and evidence-based recommendations for optimization of patient outcomes and reduction of perioperative complications.

## Review

Materials and methods

For this review, an in-depth literature search was conducted on different online databases, such as PubMed, Scopus, Web of Science, and Google Scholar, for published and peer-reviewed literature that focused on perioperative anesthesia management and guidelines in the United States. Thus, to aptly identify the guidelines and studies, we used Boolean operators with a concrete combination of various Medical Subject Headings (MeSH) terms that included "anesthesia", "cardiovascular disease", "analgesia", "perioperative care", "risk assessment", and "hemodynamic monitoring". Moreover, the search strategy entailed two phases and used the Preferred Reporting Items for Systematic Reviews and Meta-Analyses (PRISMA) guidelines in the selection and subsequent inclusion of articles for the review. The first phase involved the performance of independent screening of both the title and abstract of every article retrieved. This was done independently by two researchers. Articles with insufficient abstract data were retained for full-text screening before exclusion decisions were made. However, the researchers included articles that had insufficient abstract data with titles relevant to the review in the second phase which entailed full-text screening. In the second phase, full-text screening was conducted based on inclusion and exclusion criteria. Any potential disagreement was resolved through a third researcher who was tasked with making the ultimate decision on whether the disputed article should be included or excluded, with the decision being arrived at through consensus and consultations.

Study inclusion and exclusion criteria

This study’s inclusion criterion included original studies, like cross-sectional design studies, prospective cohort studies, and randomized controlled trials (RCTs), focusing on perioperative anesthesia management in CVD patients and guidelines in the USA. For this systematic review, only guidelines and studies that have critically assessed existing guidelines were included, as they offered primary data pertinent to the study period and findings. Moreover, only English-language literature and guidelines published in the last 20 years were included. The studies excluded were various types of editorials, opinion pieces, and narrative reviews. The evaluation of titles and abstracts of the articles was followed by the extraction of information from the eligible studies based on the general study attributes, including the authors' names, publication year, method of sampling employed, and the study population characteristics, including the gender, age, race, and sample size, and the type and duration of intervention, and the main findings. 

Results

**Figure 1:**
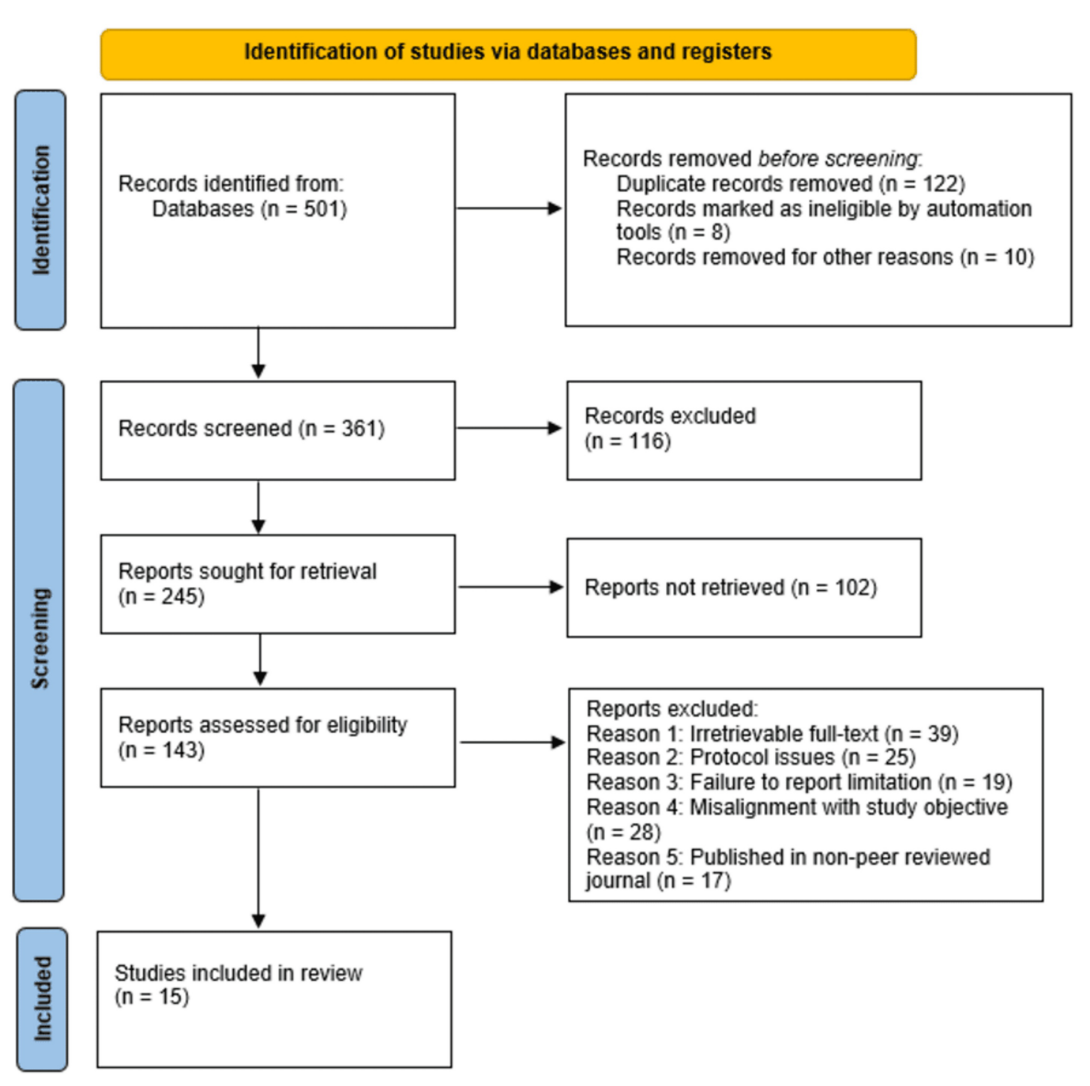
PRISMA flow diagram indicating the literature selection process for this review Records excluded (n = 112): Reasons include the failure of the study to address the research questions and populations of interest, animal-based studies, and non-primary research studies. Reports not retrieved (n = 102): Reasons include access restrictions, retrieval errors, including broken links and missing files, and withdrawn/retracted studies. n: number of studies/articles; PRISMA: Preferred Reporting Items for Systematic Reviews and Meta-Analyses (PRISMA)

The database search conducted yielded a total of 501 studies. After the removal of 140 studies (122 duplicates, eight ineligible articles, and 10 studies for other reasons), the remaining 361 studies were subjected to screening of titles and abstracts, which led to the exclusion of 116 studies due to factors that included irrelevance to the study topic, as the abstracts showed divergent objectives from the study. Following the screening, 245 studies were sought for retrieval, out of which 102 studies were not retrieved. This led to 143 studies being assessed for eligibility, which led to the exclusion of 128 studies for various reasons, including irretrievable full-text (39); protocol issues (25); failure to report limitation (19); misalignment with study objective (28); and published in a non-peer-reviewed journal (17). As a result, 15 studies [3-5, 8-10, 13, 15, 20, 23, 24, 34-37] met the inclusion criteria and were reviewed. The literature selection process for this review conducted using PRISMA guidelines is presented in Figure 1 below. Additionally, a summary of the findings of the studies included in this systematic review can be found in Table 1 below.

**Table 1:** Summary of the findings of studies included in this systematic review ACC: The American College of Cardiology; AHA: American Heart Association; ESC: The European Society of Cardiology; ESA: the European Society of Anesthesiology; ASA: American Society of Anesthesiologists; CVD: cardiovascular disease; POISE: Perioperative Ischemic Evaluation

Study/Year/citation	Study design	Findings
Fleisher et al., 2014 [3]	Clinical practice guidelines	This study developed the ACC/AHA guidelines for perioperative cardiovascular assessment in patients undergoing non-cardiac surgeries, stressing the need for risk stratification alongside personalized patient management.
Smilowitz and Berger., 2016 [4]	Meta-analysis	This study evaluated the various perioperative management strategies aimed at reducing potential cardiovascular events, underlining the significance of preoperative assessment and optimization of care.
Alwardt et al., 2005 [5]	Meta-analysis	This study evaluated the various anesthesia practices in cardiac surgeries, by concentrating on aspects that include drug selection and their potential physiological effects.
Munn et al., 2020 [8]	Methodological review	This study introduced and focused on a critical appraisal tool for assessing the quality of case series studies with regard to evidence synthesis.
Smith et al., 2023 [9]	Clinical practice guidelines	This study provided comprehensive guidelines for general anesthesia practices customized to both the patient's and surgeons' needs.
Priebe, 2011 [10]	Evidence-based report and review	This study advocated for the use of an evidence-based and personalized approach in perioperative cardiac management, particularly for non-cardiac surgeries.
American Society of Anesthesiologists, 2014 [13]	Clinical practice guidelines	The guideline provides various recommendations for the perioperative care of patients with obstructive sleep apnea, which is a condition capable of complicating anesthesia management in CVD patients.
Dobson et al., 2023 [15]	Clinical practice guidelines	This study evaluated existing anesthesia guidelines and incorporated modern practices to ascertain safety and efficiency.
Thompson et al., 2024 [20]	Clinical practice guidelines	This study presented updated guidelines for use in perioperative cardiovascular patient care, in addition to addressing the various advancements with regard to the management of non-cardiac surgeries.
Devereaux et al., 2006 [23]	Randomized controlled trial (RCT)	This study developed and utilized the POISE trial that the authors used in assessing the impacts of metoprolol on perioperative ischemic results in relation to non-cardiac surgery.
Smith Jr. et al., 2011 [24]	Clinical practice guidelines	The study updated the ACC/AHA secondary prevention guidelines used in the treatment and management of atherosclerotic vascular disease, stressing on the various risk reduction approaches.
Song et al., 2017 [34]	Evidence-based report	This study evaluated the implications of dual antiplatelet therapy's implications in non-cardiac surgeries, stressing on the importance of anesthetic considerations.
Pepine and Nichols, 2002 [35]	Evidence-based report	This study evaluated the pathophysiology of chronic ischemic heart disease and identified the major mechanisms affecting the outcomes.
Fahlenkamp et al., 2014 [36]	Clinical practice guidelines	This study offered effective ASA guidelines for the management of obstructive sleep apnea patients during perioperative care.
MacIntyre and Scott, 2020 [37]	Evidence-based report	This study provided a number of evidence-based recommendations for severe pain management within surgical contexts.

Data Collection Process

Data collection was performed under the supervision of the authors. Potential disputes and discrepancies were solved through consultations and discussions. However, in case the dispute persisted, a third reviewer was required to act as an adjudicator, with the decision being made on a majority agreement basis. Further, the extraction of data regarding the study author, year of publication, study location, the screening tools used, study sample size, and response rate was performed independently by the author using a standard data extraction format. The quality of the included studies was assessed using the Joanna Briggs Institute (JBI) quality assessment tool [8]. Consequently, all publications were scored using the frequency scale with yes, no, unclear, and inapplicable responses. Every study’s general quality score was calculated based on the overall positive scores awarded.

Data Analysis

The study attributes, such as the research design utilized, the study sample size, the publication year, and the study location, were used in assessing the potentially heterogeneous sources.

Quality Assessment

The JBI quality assessment disclosed that included guidelines and references indicated higher methodological rigor as a result of their definite inclusion criteria, well-thought-out recommendations, and increased involvement of multidisciplinary panels. Particularly, guidelines from Fleisher et al. (2014) [3], Smith et al. (2023) [9], the American Society of Anesthesiologists (2014) [13], Dobson et al. (2023) [15], Thompson et al. (2024) [20], Smith Jr. et al. (2011) [24], Fahlenkamp et al. (2014) [36], and MacIntyre and Scott (2020) [37] have offered clear recommendations, revealed conflicts of interest, and involved various expert panels. Nevertheless, a number of recommendations were dependent on expert opinion, which increased the possibility of biases. Still, among the RCTs, the study by Devereaux et al. 2006 [23] (Perioperative Ischemic Evaluation (POISE) Trial) has shown stronger methodological quality, with appropriate randomization and distribution concealment, as well as blinding, thereby ascertaining dependable statistical analysis and outcome measurements. Also, cohort studies such as Smilowitz and Berger (2016) [4] and Priebe (2011) [10] have efficiently controlled for major confounders despite having limitations resulting from their observational nature, such as potential selection biases within the retrospective analyses. Lastly, review studies, including Alwardt et al. (2005) [5], Munn et al. (2020) [8], Song et al. (2017) [34], and Pepine and Nichols (2002) [35] have offered valuable literature summaries in addition to having distinctive review questions despite lacking systematic methodologies, which makes them increasingly vulnerable to author bias. In general, the included guidelines and studies’ qualities varied between moderate and high quality, with stronger methodologies being realized in the guidelines and RCTs, whereas observational studies and reviews had intrinsic limitations impacting objectivity and causality.

Discussion

Anesthetic Agents and Choice of Technique

Regarding anesthetic agent and technique choice, it is noteworthy that four key types of anesthesia exist which include general anesthesia (intravenous or volatile anesthesia), local anesthesia, regional anesthesia (including peripheral nerve block and neuraxial blockade), and monitored anesthesia care (MAC; including sedation with/without local anesthesia) [9]. Normally, anesthesiologists regularly utilize a combination of anesthetic class agents. It is noteworthy that among the volatile agents widely used in CVD patients are isoflurane, desflurane, and sevoflurane, which are mostly preferred for their myocardial protective effects, especially their aptitude to myocardial oxygen demand reduction, induction of ischemic preconditioning, and hemodynamic stability maintenance [1-5, 10-21]. However, specific volatile anesthetics might not be suitable for every CVD patient. For instance, it is recommended that desflurane should be used carefully in CVD patients with uncontrolled hypertension, as well as those at a higher risk of tachycardia as a result of its potential to induce sympathetic activation [1-5, 10-21]. Still, CVD patients with chronic left ventricular dysfunction might not effectively tolerate desflurane and isoflurane’s vasodilatory effects, unlike sevoflurane, which is often well-tolerated and is preferred in most cardiac surgeries [10-21]. Moreover, Alwardt has asserted that, owing to their cardiodepressive effects, the agents are often used in lower dosages together with other intravenous anesthetics capable of generating the desired hypnosis [5]. Aside from muscle relaxants, analgesics, including opiates, are commonly used to blunt hemodynamic responses, particularly for highly stimulating parts of cases, including sternal opening and intubation. Further, it is recommended that, in managing anesthesia, especially general anesthesia, during cardiac surgery, efforts should be made to effectively prevent ischemia, maintain stable hemodynamics, and preserve myocardial functions [5]. Further, AHA/ACC guidelines have recommended the use of total intravenous anesthesia or any volatile anesthetic agent in CVD patients, even as the anesthesia choice is determined through factors other than concerns of myocardial ischemia [3]. Also, perioperative epidural analgesia has been recommended for reducing preoperative cardiac event incidence, particularly in patients with a hip fracture [3].

Perioperative Risk Assessment

Regarding perioperative risk assessment and stratification, it is noteworthy that perioperative assessment remains the cornerstone of perioperative management, especially in persons with CVD. However, the perioperative management of anesthesia in CVD presents various complex challenges as a result of the different and normally chronic comorbid conditions linked to cardiac pathology [10]. As such, the guidelines developed by both ACC and AHA have stressed the need for an in-depth and comprehensive assessment that incorporates the patient’s medical history, targeted investigations/interventions, and physical examination [10]. As such, one of the recommended tools for perioperative risk assessment and stratification is the Revised Cardiac Risk Index (RCRI), which has been specifically developed for conductive perioperative risk assessment in non-cardiac surgeries.

The AHA/ACC guidelines stipulate that CVD patients undergoing risk stratification surgery before cardiac surgical procedures and whose assessment necessitates coronary artery bypass graft surgery have to undertake coronary revascularization prior to the performance of the high-risk cardiac surgical procedure [3]. Thus, the AHA/ACC guidelines have recommended that patients who need coronary artery bypass graft (CABG) prior to undergoing high-risk cardiac surgery should undergo revascularization before the procedure [3]. This recommendation has been categorized as Class I with a B level of evidence. Moreover, it is recommended that the cumulative morbidity and mortality risks of cardiac procedures and coronary revascularization be carefully assessed in relation to the general health, prognosis, and functional status of the patient.

Additionally, the AHA/ACC guidelines have recommended a preoperative noninvasive risk stratification aimed at assessing the existence of myocardial ischemia in CVD patients who have been identified as having lower functional capacity and with elevated risk for non-cardiac surgery, given that stress echocardiography and abnormal myocardial perfusion imaging tests can be used in predicting the potential for postoperative cardiovascular events [4]. It is noteworthy that the non-invasive investigations are mainly performed in patients with established and high-risk cardiovascular disease, especially older persons. Pre-operative assessments, including investigation and treatment, may additionally be improved by novel methods that include coronary computed tomographic angiography. Moreover, intensive medical management, mainly high-intensity statin therapy, has been found to minimize perioperative cardiovascular risks. Further, non-invasive anatomic testing using coronary computed tomographic angiography should be conducted prior to the performance of a non-cardiac surgery is a promising approach that requires further study. Thus, coronary computed tomographic angiography (CCTA) is liable to provide increasingly precise exclusion of significant coronary artery disease; however, in instances where atherosclerosis has been detected, the perioperative management’s impact will be debated, given that the existing recommendations are mostly based on low-level evidence [4-7]. Although the detection of coronary atherosclerosis might aid in guiding perioperative management, limited evidence is available to support the altering of surgical decisions. In this regard, efforts are currently underway to identify novel approaches to reduce perioperative cardiovascular events in high-risk patients and to manage patients who develop postoperative myocardial injury [4].

Additionally, the functional capacity assessment is conducted with the objective of evaluating the metabolic equivalents (METs) in order to ensure that the patient has the necessary aptitude to engage in everyday activities, and this is considered a key predictor of perioperative risk. Metabolic equivalents are vital for the evaluation of functional capacity and for guiding the perioperative risk stratifications in patients with CVDs undergoing non-cardiac surgeries [11-14]. Thus, the ACC/AHA guidelines recommend that individuals with ≥4 METs may characteristically proceed with surgery devoid of additional testing, even as individuals with <4 METs should be subjected to further evaluation reliant on surgical risks [4]. Still, individuals with severely limited METs (<2) have a high perioperative risk, which necessitates optimization and alternative management interventions [4, 11-14]. The functional capacity assessment is performed, particularly in preoperative evaluations, mainly to approximate the risk of perioperative complications and death in CVD patients. The preoperative functional status is notably the most significant predictor of perioperative outcomes. For instance, lower exercise tolerance has been linked to poor perioperative outcomes [11, 12]. As such, the objective of preoperative evaluation of functional capacity is mainly to predict the aptitude of an individual to increase oxygen delivery during the perioperative period. In emergency situations, perioperative care should prioritize hemodynamic monitoring and cardioprotective measures. As such, tools such as the Duke Activity Status Index (DASI) and the RCRI are recommended for use in risk assessment and decision-making, given their effectiveness in evaluating functional capacities and guiding perioperative strategies [4, 11, 12]. Even though perioperative cardiac optimization can be restricted to emergencies, the incorporation of METs into the decision aids in the stratification of risks and improvement of patient care outcomes.

Still, the guidelines developed by the ACC/AHA have all included cardiopulmonary fitness approximations as a means of estimating functional capacity [3], prior to the performance of major non-cardiac surgeries. The guidelines recommend the use of subjective assessment in the estimated functional capacity by asking patients questions regarding their routine activities, including walking and exercises [13, 14]. That is, according to the Guidelines to the Practice of Anesthesia: Revised Edition 2023, the perioperative assessment needs to be performed by looking into aspects that include the patient’s medical history and examination and by focusing on factors that include medication reviews, functional status assessment, and recent cardiac events [15]. The guidelines have recommended additional cardiac investigations for CVD cases found to be high-risk. Further, for effective and better anesthesia and patient care outcomes, the guidelines have recommended the provision of intraoperative care through closer monitoring, fluid management customized to the patient’s cardiac status, and the use of cardio-protective anesthetic techniques [15]. Consequently, with regard to postoperative monitoring, the guideline has emphasized increased vigilance for complications that include arrhythmias and myocardial ischemia, which is important, particularly within the step-down or intensive care contexts [15]. Still, concerning postoperative monitoring, increased monitoring for various complications, including myocardial ischemia and arrhythmias, is important, especially within the intensive care and step-down contexts. Owing to the subclinical ischemia potential, characteristically present without the classic chest pain, perioperative electrocardiogram (EKG) surveillance alongside serial troponin measurements is important for timely detection and interventions [15]. The approach ascertains that potential ischemic events, such as ones that manifest as angina equivalents during the postoperative period, are detected, thereby enhancing patient care outcomes and safety.

Still, the guidelines have recommended comprehensive cardiac testing only in instances where the outcomes are highly prone to affect perioperative management. Additionally, it is recommended that multi-disciplinary teams comprising anesthesiologists, cardiologists, and surgeons be constituted, especially in instances where the CVD cases involved have been assessed and determined to be high risk [8, 16]. Such teams have further been recommended by various guidelines, including the Guidelines to the Practice of Anesthesia: Revised Edition 2023, which has additionally highlighted the need for collaborations between anesthesiologists and cardiologists for customized perioperative planning. The guideline has stressed the need to utilize evidence-based protocols, as this enables effective mitigation of various cardiovascular risks and the optimization of patient outcomes [8, 15]. Among the notable examples recommended in the guidelines is the use of various standardized preoperative risk evaluation tools that include the ACC/AHA guidelines for perioperative cardiovascular assessment and the RCRI [15]. These can be implemented using multidisciplinary collaborations, as they permit the execution of individualized interventions, including statin therapy, beta-blockade, and various personalized hemodynamic monitoring strategies that are based on patient-specific risk factors. Such measures are vital to the optimization of patient outcomes through the reduction of perioperative cardiac complications. Nevertheless, in the assessment of the various risks and advantages of the perioperative intervention and management approaches in CVD patients, it is recommended that the risks linked to non-cardiac surgeries be individualized, as this will enable the anesthesiologists to aptly exercise judgment and evaluate the perioperative surgical risks alongside the need for additional assessment [8]. The effective management of CVD patients calls for the identification of potential risk factors, medical therapy, preoperative assessment and optimization, effective monitoring, and the selection of correct anesthesia and anesthetic techniques [15]. Moreover, the Guidelines to the Practice of Anesthesia: Revised Edition 2023 have maintained that, for perioperative anesthesia management in CVD patients, patient safety should be established through comprehensive risk evaluation alongside cardiac function optimization before the surgical procedure.

Perioperative Planning

Regarding perioperative anesthesia management, it is noteworthy that every anesthetic drug and technique has acknowledged cardiac effects that have to be taken into consideration during the perioperative planning [5, 17]. At present, no appropriate myocardium-protective anesthetic technique exists [3, 4, 10]. As such, the anesthesia choice alongside intraoperative monitoring is often left to the anesthesia team, which is tasked with considering the requirement for postoperative ventilation, sympathetic blockade, the procedure’s dermatomal level, and the cardiovascular effects. In recommending monitored anesthesia, which entails the supplementation of the local anesthesia through intravenous sedation and analgesia, Meng et al. maintain that the application of the technique enables the avoidance of unfavorable effects of either the neuraxial or general techniques; however, this has never been established by any study [18]. Further, in their study, Meng et al. have highlighted the benefits of MAC, especially with regard to the mitigation of the negative hemodynamic effects of general and neuraxial anesthesia [18]. Thus, the findings of the study conducted by Meng et al. [18] are aligned with the findings of earlier studies that have emphasized hemodynamic stability offered by MAC, particularly in cardiovascular patients classified as high-risk [1, 9, 10, 15]. Nevertheless, this has remained debatable, given that, at present, no RCTs have conclusively proven the supremacy of MAC over other diverse methods with regard to myocardial protection. Additionally, the failure to produce complete analgesia or local anesthesia may result in increased levels of stress response, as well as myocardial ischemia, particularly in instances of insufficient dosing and incorrect methods, drug interactions, usage of short-acting anesthetics, and increased sensitivity and tolerance to pain and higher analgesia thresholds that make it increasingly challenging to realize sufficient pain control using standard doses.

Several guidelines and studies have recommended anesthetic considerations along with effective intraoperative management [3, 4, 19]. Every medication and anesthetic technique has various known cardiac effects that must be considered by the anesthetist during the preoperative planning for CVD patients [3, 4]. Nevertheless, at present no single best myocardium-protective anesthetic technique exists [19]. Moreover, anesthesia teams also experience difficulties in balancing cardiovascular stability with sympathetic blockade. For example, excessive sympathetic blockade attributable to neuraxial anesthesia may result in significant hypotension in patients undergoing vascular surgery (major), which necessitates vasopressor support [17-20]. On the contrary, in severe coronary artery disease patients, general anesthesia is likely to increase the consumption of myocardial oxygen, which poses significant ischemic risks [17-20]. Under such instances, a multimodal strategy that integrates aspects of regional blocs, MAC, and individualized sedation is recommended, as they are liable to optimize the outcomes.

Further, the administered oxygen concentration has additionally been studied during the perioperative duration, with a number of studies assessing the impact of 30% against 80% of the fraction of inspired oxygen in relation to myocardial infarction and injuries in the course of surgery [20]. Two RCTs have independently disclosed that there was no correlation between oxygen concentration and increment in myocardial injury risk within three days or following NT-proBNP postoperative release [20-24]. The findings of the studies have corroborated the outcomes of previous retrospective analyses comprising 1,617 patients who underwent surgical procedures and found that there were no correlations between the increment in oxygen concentration and myocardial injury, 30-day mortality, and cardiac arrest incidence [20-24]. Additionally, various studies have assessed the perioperative effects of oxygen concentration, especially in the reduction of infections within the surgical sites, as well as enhancing tissue oxygenation. Nonetheless, such concerns have remained, particularly in relation to possible hyperoxia-induced vasoconstriction and oxidative stress, which are likely to counteract such benefits [15-20]. Among the notable limitations of such studies are heterogeneity in patient populations, smaller sample sizes, and divergences in surgical contexts that have made it increasingly difficult to draw general recommendations. Regardless of such concerns and limitations, existing guidelines, including the American Society of Anesthesiologists guidelines, have recommended personalized oxygen titration as opposed to a one-size-fits-all approach.

Pharmacological Therapy

The AHA/ACC Guidelines on Perioperative Cardiovascular Evaluation and Management of Patients Undergoing Non-Cardiac Surgery have recommended the perioperative utilization of beta blockers, owing to their correlations to significant reductions in cardiac events, particularly in individuals with coronary artery disease and at high cardiovascular risk [3, 24]. Cautious initiation of beta blockers should be conducted to avert potential bradycardia and perioperative hypotension, even as it is recommended that beta blockers should not be commenced acutely prior to surgery but have to be continued in individuals on chronic therapy [24]. Beta-blockers have been acknowledged to aid in the mitigation of surgical cardiac stress through the reduction of blood pressure, myocardial contractility, and heart rate, even as they have also been acknowledged for their roles in the reduction of myocardial infarction risks in patients undergoing non-cardiac surgeries [3]. However, a limited amount of data has supported the efficiency of preoperative beta-blocker administration as a means of reducing surgical death risks among CVD patients. To tackle the risks linked to beta-blockers, including bradycardia and stroke, various risk mitigation strategies are recommended. For instance, careful patient selection should be conducted, close monitoring (perioperative) of the patient’s blood pressure and heart rate, and performance of gradual titration prior to surgery [3]. Furthermore, it is recommended that practical adjustments, including the withholding of beta-blockers, particularly in patients with uncompensated heart failure and significant bradycardia, be made in addition to consideration of alternative agents [3]. Additionally, the guidelines have indicated the existence of consistent and palpable associations between the administration of beta blockers and adverse outcomes that include stroke and bradycardia [3]. Further, it is noteworthy that the recommendations made by the guidelines are increasingly consistent, even with the exclusion of the DECREASE studies [21, 22] and the POISE Study [23]. The exclusion of POISE study and the DECREASE studies were excluded as a result of potential biases capable of affecting their findings and reliability. For instance, the DECREASE studies faced concerns regarding methodological flaws, even as the POISE study, notwithstanding being a bigger RCT, was criticized for the use of a high-dose beta-blocker regimen that not only did not reflect characteristic clinical practice but has been linked to a higher risk of stroke. In this regard, it is noteworthy that the study's exclusion did not proffer any significant effects on the risk estimates and the benefits. In this light, the guidelines have stipulated that the beta blockers need to be continued, especially in individuals undergoing surgical procedures and who have been chronically on beta blockers [24]. The guidelines further recommend continuation of the beta-blockers in instances where they are well-tolerated by the CVD patients receiving them for various longitudinal reasons, especially in instances where the provision of the longitudinal treatment is based on guideline-directed medical therapy (GDMT), as for myocardial ischemia [3]. The benefits and risks associated with perioperative beta blockers have been acknowledged to be increasingly favorable to CVD patients who have an intermediate or high risk for myocardial ischemia, as noted on the preoperative stress assessment [25-27]. However, according to Smith et al., the decision to commence the use of beta blockers needs to be inspired by the determination of whether the CVD patient is at high risk for stroke [24], along with the determination of the existence of relative contraindications that include uncompensated heart failure. Existing observational data have further indicated that patients who seem to gain from beta-blocker use within the perioperative contexts include those with ≥3 RCRI risk factors [23, 27, 28]. In this regard, the guidelines maintain that commencing beta-blockers early enough in advance of the surgical procedure data to enable the assessment of tolerability and clinical efficiency. Building on the focus on perioperative use of beta-blockers and the associated benefits and risks, it is also important to consider the various roles of other notable cardiovascular medications, including statins, angiotensin-converting enzyme (ACE) inhibitors, and angiotensin-receptor blockers (ARBs), in the optimization of surgical outcomes for the patients.

Different guidelines have stipulated the continuation of statin therapy for CVD patients presently on statins and also scheduled for non-cardiac surgical procedures [29-31]. Moreover, perioperative statin use initiation has been recommended for CVD patients undergoing vascular surgeries and should also be considered in individuals with clinical indications as per GDMT and undergoing various high-risk procedures [32]. Thus, regarding their pleiotropic effects beyond the acknowledged lipid-lowering properties, particularly in vascular surgery patients, statins have well-established pleiotropic properties that extend beyond the reduction of cholesterol. These properties make statins beneficial for vascular patients, as they improve endothelial functions through bioavailability of nitric oxide, reduction of thrombosis and inflammation risks, stabilization of atherosclerotic plaques, modulation of the immune responses, provision of renal protection, and lowering perioperative atrial fibrillation incidence, which are aspects that have significant effects on improvement of vascular surgery outcomes [29-32]. Despite the stated beneficial pleiotropic effects, in high-risk populations, statin therapy has been marked by controversies and limitations, including concerns regarding potential adverse effects, including myopathy, dose adjustments, potential negative effects, liver dysfunction, and optimum initiation timing [29-32]. Therefore, additional studies are required to refine existing guidelines for the use of statins in vascular surgery patients beyond the stated lipid-lowering attributes. Additionally, perioperative ACE inhibitors and ARB continuation are recommended and reasonable [33]. Though several guidelines have recommended the suspension of ARBs and ACE inhibitors preoperatively as a result of the potential risk of distributive shocks and intraoperative hypotension, the decision should be personalized, especially for heart failure and hypertension patients, in which sudden discontinuation is likely to result in hemodynamic instability [29-33]. It has also been disclosed that withholding such medications is liable to reduce intraoperative hypotension; however, postoperative resumption is recommended when clinically feasible to effectively avert adverse cardiovascular events [29-33]. In instances where the ARBs and ACE inhibitors are withheld prior to the surgical procedure, a restart is recommended to commence sooner as clinically practicable postoperatively. Moreover, at present, the perioperative ACE inhibitors and ARBs use in hypovolemia and pre-existing renal insufficiency patients is debatable owing to the risks, including acute kidney injury (AKI) and hypotension. Thus, postoperative hypotension, normally exacerbated by anesthesia-linked vasodilation, is likely to increase morbidity, including renal dysfunction and myocardial ischemia [29, 33]. As a result of these concerns, existing guidelines have recommended the withholding of such agents prior to surgery and delaying the reintroduction up to a time when hemodynamic stability is realized [33]. Among the notable safer reintroduction strategies is the assessment of hemodynamic stability to ascertain that the patients are not only free from persistent ‘hypotension but also euvolemic [29, 33]. Progressive dose escalation, which entails restarting with lower dosages and titrating slowly, as well as monitoring the renal functions by tracking the estimated glomerular filtration rate (eGFR) and serum creatinine to detect AKI, has been recommended as an appropriate reintroduction strategy [29-33]. The other recommended reintroduction strategies include the use of an individualized approach, given that high-risk patients are likely to gain from earlier reintroduction even as low-risk patients might need prolonged withholding [29-33]. Also, alternative agents, including beta-blockers and calcium channel blockers, should be considered in instances where the safe resumption of ARBs and ACE inhibitors is impossible.

For CVD patients undergoing emergency non-cardiac surgeries within four to six weeks of drug-eluting or bare-metal stent implantation, dual antiplatelet therapy (DAPT) should continue unless the bleeding risk outweighs the benefit of preventing stent thrombosis [3, 34]. Moreover, individuals receiving coronary stents are required to undergo surgery that mandates P2Y12 platelet receptor-inhibitor therapy discontinuation, and aspirin should be continued when possible with the P2Y12 platelet receptor-inhibitor being restarted after the surgery [3]. According to Song et al., perioperative antiplatelet therapy management is mainly established through consensus between the patient, anesthesiologist, surgeon, and cardiologist, tasked with weighing the comparative risk of bleeding with the risk of stent thrombosis [34]. The multidisciplinary approach that involves anesthesiologists, cardiologists, and nephrologists is recommended as it will aid in optimizing management and minimizing potential complications. Moreover, perioperative DAPT management in CVD patients undergoing non-cardiac surgeries requires the effective balancing of bleeding and thrombotic risks [3-6, 34]. The decision on suspension or continuation of DAPT is dependent on various factors, including stent type, surgical urgency, and time from the percutaneous coronary intervention (PCI), as well as patient-specific risks, including prior infarction and diabetes. In emergency surgical cases, particularly in instances of <4-6 weeks post-PCI, it is recommended that DAPT continuation occur owing to the elevated risk of stent thrombosis, even as in urgent surgery involving cases of one to six months post PCI, it is recommended that the P2Y12 inhibitor be discontinued even as aspirin should be maintained [3-6, 34]. Moreover, in cases of >6 months post-PCI, elective surgeries enable safer discontinuation of P2Y12 inhibitor, and aspirin continuation is recommended unless contraindicated [34]. Early suspension of DAP, during the initial month post PCI, significantly increases stent thrombosis and reinfarction risks, particularly in high-risk individuals with recent infarction, diabetes, and complex coronary lesions [34]. Other factors, including vessel size, type of infarction, and angioplasty timing, also influence the risk of thrombosis. Further, for high-risk individuals in need of urgent surgery, bridging using short-acting intravenous antiplatelet agents that include tirofiban and cangrelor might be considered an appropriate alternative in the instance of P2Y12 inhibitor suspension.

Perioperative Pain Management

Guidelines by AHA/ACC and Pepine and Nichols have recommended and advocated for perioperative pain management and have maintained that patient-controlled epidural or intravenous anesthesia/analgesia should be used in the effective management of postoperative pain when indicated [3, 35]. This recommendation has been supported by a number of studies that have suggested that effectual pain management aids in the reduction of hypercoagulability and postoperative catecholamine surges [18, 36]. Moreover, effective management of perioperative pain aids in preventing and mitigating stress responses to surgeries that may initiate increased release of catecholamines, including norepinephrine and epinephrine [18, 35, 36]. This, in turn, results in increased vasoconstriction, blood pressure, and heart rate, aspects that have been acknowledged to exacerbate cardiovascular risks. Moreover, poor pain management has also been acknowledged to promote a prothrombotic state through increased aggregation of platelets and various inflammatory mediators, which increases the risk of myocardial ischemia and thrombosis [18, 35, 36]. In agreement with the guidelines recommendations, AHA/ACC has maintained that, in addition to pain management being basic in relation to the right to health, insufficiently treated pain in CVD patients contributes significantly, and in infrequent instances, results in mortality [3]. Moreover, uncontrolled severe pain additionally increases the chronic pain incidence, thereby imposing some level of disability and suffering that might last for several years [37]. Also, it has been disclosed that poorly managed perioperative pain may result in nearly a 30% increment in the risk of myocardial infarction resulting from increased hypercoagulation and elevated sympathetic activation [3, 35-38]. Further, an increase in mortality rates has been reported among patients with severe pain attributable to a response to chronic stress that exacerbates the pre-existing cardiovascular disease [3, 35-38]. The effects of pain management in relation to the outcomes have further been stratified based on patient demographics and severity of the disease, even as elderly patients with chronic heart disease are at a high risk of developing complications. It is in this light that recommendations have been made to ensure that the first postoperative patient care is offered by anesthesiologists, including monitoring of the patient's hemodynamic stability, medical plan optimization to avert potential negative cardiovascular events, and provision of individualized pain management based on the patient's condition, and this is followed by surgeons and other healthcare professionals, as this has been acknowledged to enhance patient care outcomes [38]. Moreover, neuraxial anesthesia has been recommended by AHA/ACC guidelines with regard to postoperative pain management, given its effectiveness, particularly in CVD patients who have undergone abdominal aortic surgery aimed at reducing the incidence of perioperative myocardial infarction [4].

## Conclusions

In conclusion, anesthesia entails more than the observation of an unconscious patient. In this regard, the four key anesthesia components must not only be provided but additionally managed with care, given the potential adverse effects of anesthesia. Thus, the most rational approach to the induction and subsequent maintenance of anesthesia requires a combination of different anesthetics/agents, including intravenous and inhaled anesthetics, with definite actions. Despite the lack of universal agreement among anesthesiologists on the provision, development, and use of a general protocol, careful consideration of every type of anesthesia and the related effects on patients with CVD is important to the provision of safer and appropriate care to such patients. Moreover, given the importance of perioperative anesthesia management to patient care outcomes, the various aspects of preoperative, intraoperative, and postoperative factors, including endocrine, hemodynamic, surgical care issues, inflammatory responses, metabolic, surgery duration, hypovolemia, pain, and pulmonary dysfunction, should be considered, owing to their significant impact on the overall patient care outcomes. Lastly, based on the review findings, it is recommended that prospective studies evaluate individualized anesthetic approaches and protocols based on specific cardiovascular diseases, including arrhythmias, heart failure, and ischemic heart disease, to minimize adverse effects. Still, future studies should evaluate various advanced intraoperative monitoring methods, including artificial intelligence (AI)-based predictive analytics and real-time hemodynamic evaluations, as these techniques are liable to improve patient safety.
